# Saliva collection via capillary method may underestimate arboviral transmission by mosquitoes

**DOI:** 10.1186/s13071-022-05198-7

**Published:** 2022-03-24

**Authors:** A. Gloria-Soria, D. E. Brackney, P. M. Armstrong

**Affiliations:** Center for Vector Biology & Zoonotic Diseases, Department of Environmental Sciences, The Connecticut Agricultural Experiment Station, 123 Huntington St., New Haven, CT 06504 USA

**Keywords:** Arbovirus, Transmission, Vector, Proxy, Saliva

## Abstract

**Background:**

Arthropod-borne viruses (arboviruses) impose a major health and economic burden on human populations globally, with mosquitoes serving as important vectors. Measuring the ability of a mosquito population to transmit an arbovirus is important in terms of evaluating its public health risk. In the laboratory, a variety of methods are used to estimate arboviral transmission by mosquitoes, including indirect methods involving viral detection from mosquito saliva collected by forced salivation. The accuracy of indirect methods to estimate arbovirus transmission to live animal hosts has not been fully evaluated.

**Methods:**

We compared three commonly used proxies of arboviral transmission, namely, the presence of virus in mosquito legs, in salivary glands (SG) and in saliva collected in capillary tubes using forced salivation, with direct transmission estimates from mosquitoes to suckling mice. We analyzed five vector-virus combinations, including *Aedes aegypti* infected with chikungunya virus, West Nile virus and Zika virus; *Culex quinquefasciatus* infected with West Nile virus; and *Aedes triseriatus* infected with La Crosse virus.

**Results:**

Comparatively, the methods of detecting virus infection in mosquito legs and in SG were equally accurate in predicting transmission. Overall, the presence of virus in mosquito legs was a more accurate predictor of transmission than the commonly implemented viral detection method using forced salivation into a capillary tube, and was subject to less technical variation.

**Conclusions:**

These results suggest that, in general, forced salivation methods tend to underestimate virus transmission, and they provide confidence in the use of mosquito leg screens to evaluate the transmission potential of a mosquito population.

**Graphical Abstract:**

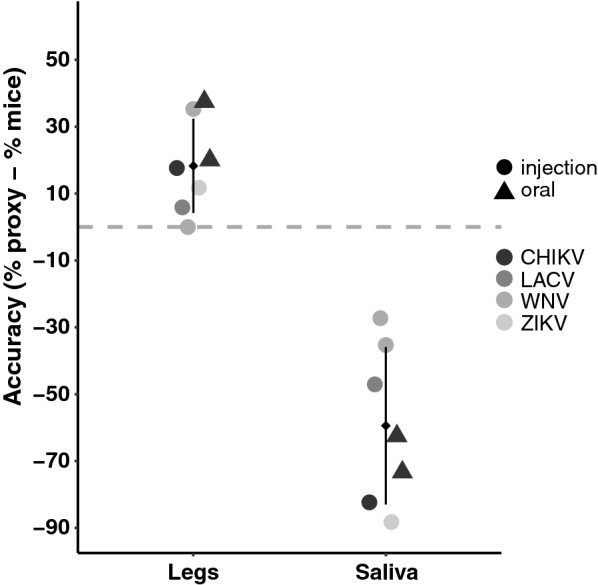

**Supplementary Information:**

The online version contains supplementary material available at 10.1186/s13071-022-05198-7.

## Background

Arthropod-borne viruses (arboviruses) impose a major public health burden on affected populations and are on the rise in both tropical and temperate regions of the world. In recent decades, chikungunya virus (CHIKV), West Nile virus (WNV) and Zika virus (ZIKV) have spread to new geographic regions, driving research on the vector competence of local mosquito populations for these and other invasive arboviruses. Vector competence is defined as the ability of an arthropod to acquire, maintain and transmit the pathogen to a vertebrate host during the blood-feeding process. In the laboratory, a variety of methods are used to estimate arboviral transmission by a mosquito vector. Direct in vivo methods involve exposing laboratory animals to infected vectors, which allows mosquitoes to engage in the natural blood-feeding process that is required for arbovirus transmission. However, in vivo methods are rarely used today due to high costs, requirements for personnel training, appropriate husbandry and containment facilities and animal permits [1]. Furthermore, the use of vertebrate hosts for these studies is limited to the availability of competent animal models for the arbovirus under study [1]. More frequently, transmission is estimated by indirect in vitro methods, which are more accessible and cost-effective but can be labor-intensive. Among the in vitro methods in current use, the capillary method of forced salivation is the most widely used assay, involving imobilization of individual mosquitoes and subsequent harvesting of their saliva for arboviral testing by inserting their proboscis in a finely drawn capillary tube or pipette tip. Another approach is to simply screen the peripheral tissues (legs, salivary glands [SG] and/or head tissue) for arbovirus infection with the underlying assumption that these measures of disseminated infection are equivalent to virus transmission [2–4].

The complexity involved in the experimental design of vector competence trials complicates comparisons across studies [5]. Nevertheless, it is worth noting that these studies often report sharp declines in the percentage of mosquitoes transmitting virus after developing a disseminated virus infection [6–9]. This could imply either a strong SG infection or an escape barrier in these vectors [10], or perhaps this is an artifact of the techniques used to assess arbovirus transmission. For example, *Aedes aegypti* and *Aedes albopictus* often demonstrate poor vector competence for CHIKV, DENV, and ZIKV in the laboratory, even though they are known to serve as efficient vectors for these arboviruses in nature [5, 11, 12]. These studies have relied on forced salivation techniques to demonstrate virus transmission, which does not allow mosquitoes to probe or feed naturally.

The indirect methods used to estimate arbovirus transmission by mosquitoes have not been fully validated, despite their widespread use in vector competence studies. In a study by Styer et al. [13], mosquitoes were found to deliver approximately 600-fold more WNV after probing on mice than recovered by a forced salivation technique (the capillary method), suggesting that some methods may underestimate arbovirus transmission to live animal hosts. Accordingly, to explore this possibility further, we compared three commonly used proxies of arboviral transmission (indirect methods), namely, the presence of virus in legs, SG and saliva, with direct transmission estimates from mosquitoes to suckling mice (“true transmission rate”) to determine the most informative and efficient proxy for arbovirus transmission in five vector–virus combinations.

## Methods

### Mosquito species

Mosquitoes were reared from eggs in plastic trays, and larvae were fed as needed with either TetraMin® tropical flakes (Tetra GmbH, Melle, Germany) or a 2% solution of 3:2 liver powder and brewer's yeast mix. Adults were kept in 30 × 30 × 30-cm cages and provided with a 10% sucrose solution *ad libidum* on a cotton ball. The *Ae. aegypti* Orlando (ORL) colony was obtained from the Agricultural Research Service of the US Department of Agriculture (USDA ARS, Gainesville, FL, USA), originally derived from field-collected mosquitoes from Orlando Florida in 1952 (Gloria-Soria et al. [14]). The *Aedes triseriatus* colony originated from mosquitoes collected in Waterford, CT (USA) in 1992. The *Culex quinquefasciatus* colony was established from mosquitoes originally purchased from Benzon Research Inc. (Carlisle, PA, USA) and is believed to have originally been derived from mosquitoes in northern Florida.

### Virus strains

CHIKV LR2006-OPY1 (GenBank accession no. KT449801.1) was obtained from the World Reference Center for Arboviruses at the University of Texas Medical Branch, Galveston, TX (USA). This strain was originally isolated from serum of a patient returning from La Réunion Island in 2006, and passed five times on Vero cell culture, once in suckling mice and once in C6/36 cells. WNV 2741-99 was isolated from *Culex pipiens* collected in Greenwich, CT (USA) in 1999 and was passaged 4 times on Vero cells. ZIKV MR766 (GenBank accession no. MW143022.1) was obtained from The Arbovirus Reference Collection from the Centers of Disease Control (Fort Collins, CO, USA). This strain was originally isolated from a sentinel rhesus monkey in 1947 at Zika Forest, Entebbe, Uganda, and repeatedly passed in mice, twice on Vero cell culture and once on C6/36 cell culture. La Crosse virus (LACV) 78V-8853 strain was isolated in 1978 from an *Ae. triseriatus* mosquito from Rochester, MN (USA) and passaged once on Vero cells, twice on suckling mice and twice more on Vero cells.

### Experimental infections

#### Intrathoracic inoculation

Female mosquitoes aged 6 to 10 days were inoculated intrathoracically with 69 nl of a virus solution prepared from a frozen viral stock to contain approximately 17 plaque-forming units (PFU) of the virus. Females were incubated at 28 °C and a 14:10 (light:dark) cycle, with free access to a 10% sugar solution from a cotton ball, for the extrinsic incubation period (EIP) described in Table 1.

**Table 1:** Details of the intrathoracic inoculation experiment

Mosquito species	Virus	Virus strain	PFU	EIP (days)	IIP (days)	References
*Aedes aegypti* [ORL]	CHIKV	L006-OPY1	17.2	6	3	[15, 34–36]
*Aedes aegypti* [ORL]	ZIKV	MR766	17	10	6	[16, 36–38]
*Aedes aegypti* [ORL]	WNV	2741-99	16.8	10	6	[19, 37, 38]
*Culex quinquefasciatus*	WNV	2741-99	16.8	7 and 10	6	[19, 37, 38]
*Aedes triseriatus*	LACV	78V-8853	18.1	7	3	[18]

EIP, Extrinsic incubation period (days of mosquito incubation post-infection); IIP, intrinsic incubation period (days of suckling mice incubation post-infection); for other abbreviations, see Abbreviations section

#### Oral infections

Female mosquitoes aged 6 to 10 days were sorted into meshed pint cups and deprived of sugar 24 h prior to the experiment. On the day of the experiment, mosquitoes were offered an infectious blood meal consisting of a 1:1 mix of freshly grown virus culture and defibrinated sheep’s blood (HemoStat Labs, Dixon, CA, USA). The viral culture was obtained by inoculating a confluent monolayer of *Ae. albopictus* C6/36 cells into a T25 flask containing 100 ul of CHIKV stock virus, followed by a 3-day incubation; the culture was harvested on the day of the experiment to prepare the mix. The blood meal was warmed at 37 °C for 45 min in an artificial membrane system lined with hog sausage casing. Fully engorged mosquitoes were retained and incubated for 12 and 15 days at 28 °C under a 14:10 (light:dark) cycle, with free access to a 10% sugar solution provided on a cotton ball.

### Transmission estimates

Immunocompetent suckling mice have been shown to develop systemic infection and viremia upon exposure to CHIKV [15], ZIKV [16, 17], LACV [18] and WNV [19]. In this study we used infection developed in post-exposure suckling mice as the “true arbovirus transmission rate” and compared this rate to transmission estimates based on mosquito tissue/saliva proxies. Litters of suckling mice (mixed sex) from pregnant CD-1 mice were obtained from the Charles River Laboratories (Wilmington, MA, USA). Procedures for handling and care of animals were approved by and performed under the Animal Care and Use Committee at The Connecticut Agricultural Experiment Station (protocol no. P28-17). Suckling mice were tattooed to track individual mice from each litter. Individual mosquitoes were randomly assigned to individual suckling mice after the corresponding EIP had elapsed (Table 1). Each mosquito was allowed to feed on a restrained mouse placed on top of a screened cage at 28 °C. Following feeding, the legs and wings of engorged mosquitoes were removed. Saliva was collected from mosquitoes by inserting their proboscis into P20 microcapillary gel-loading tips containing 5 ul of a 1:1 solution of 50% sucrose:fetal bovine serum for 1 h at room temperature. The tip contents were expelled into a tube with 50 ul of PBS-G (phosphate-buffered saline, 30% heat-inactivated rabbit serum, 0.5% gelatin). It should be noted that Miller et al. [20] have recently demonstrated that blood-feeding immediately prior to forced salivation does not affect arbovirus expectoration. SG were dissected. Abdomens, legs and SG were independently homogenized in 200 µl of PBS-G in a 2-ml microcentrifuge tube using Copperhead copper beads (Crosman Corp., Boomfield, NY, USA) and a Mixer Mill 400 (Retsch GmbH, Haan, Germany) for 30–60 s at 24 Hz. All samples were stored at − 80 °C until RNA extraction.

Mice were kept in a Thoren cage rack system (Thoren Caging Systems Inc., Hazleton, PA, USA) with HEPA filtration under a 12:12 (light:dark) cycle and observed daily until they displayed illness symptoms or for a maximum number of days after exposure, corresponding to the intrinsic incubation period (IIP) reported in Table 1. At that point, mice were euthanized, and one hind limb and brain tissue were collected, homogenized in 500 µl of PBS-G (as described above) and stored at − 80 °C until RNA extraction.

Mosquito viral dissemination after IT injection was confirmed by testing the mosquito legs via real-time PCR (RT-PCR). Infection by oral feeding was confirmed by testing the mosquito abdomen by the same method. The ability of the mosquito to achieve viral transmission was assessed via four parameters: (i) detection of virus in mosquito legs; (ii) detection of virus in mosquito SG; (iii) detection of virus in mosquito saliva; and (iv) development of systemic infection of exposed suckling mice.

### Virus detection

We maximized our ability to detect the presence of arboviruses in the samples by screening for RNA viral copies via RT-PCR. This detection method is more sensitive than quantifying infectious particles via plaque assay because for every arboviral infectious particle there are at least 10^2^–10^4^ more RNA viral particles [20–22].

RNA was extracted from 50 ul of the homogenized tissue and saliva solution using the KingFisher™ Flex Purification System (Thermo Fisher Scientific, Waltham, MA, USA) with the Mag-Bind ® Viral DNA/RNA Kit (Omega Bio-Tek, Norcross, GA, USA) and eluted in a final volume of 50 µl of 1/10X TE buffer (Tris–EDTA). A 2-μl aliquot of this eluate was then used in each 10 µl RT-PCR reaction. Presence of virus was determined in duplicate with the I-Taq™ Universal probes 1-Step-Kit (BioRad Laboratories, Hercules, CA, USA), using primers and probes described in Additional file 1: Table S1. Samples were only thawed once. Results were analyzed with the BioRad CFX Maestro™ Software for Mac 1.1. v.4.1.2 (BioRad Laboratories) using a baseline of 100. A mosquito was considered infected if viral RNA was detected in the abdomen and considered to have a disseminated infection if the legs were positive. A cycle threshold (Ct) cut-off value < 37 was used in the RT-PCR assays to consider a sample positive for WNV, LACV and ZIKV, and a Ct value < 35 was used for CHIKV; these cut-off values were based on the detection limit of our assay, established from standard curves (Additional file 1: Supplementary information; Fig. S1; Table S2).

### Statistical analyses

Proportions of positive samples were compared using a Fisher exact test [23] applying the Holm correction for multiple testing [24], as implemented by the prop.fisher.test() function in the fmsb v.0.7.1 package [25] from R v. 4.0.3. [26]. Graphs were generated in R v. 4.0.3. [26] and 95% binomial confidence intervals for each proportion were calculated with the prop.test() function available in the software core stats package. A Welch two-sample t-test was used to compare the accuracy of legs and saliva to predict transmission.

## Results

### Evaluation of transmission proxies in mosquitoes with disseminated infections

We examined the accuracy of salivary glands and saliva as proxies for arboviral transmission in multiple mosquito-virus pairings using mosquitoes infected by intrathoracic (IT) inoculation. This approach was utilized to ensure viral dissemination at the time of the transmission assay. After the corresponding EIP elapsed (Table 1), mosquitoes were allowed to feed on suckling mice, and the presence of arbovirus in legs, salivary glands and saliva was determined. The presence of virus in legs was used to confirm dissemination. The “true transmission rate” was based on the percentage of suckling mice that developed infection within the IIP, and the accuracy of each transmission proxy was determined by comparing this value to the proportion of positive samples derived from SG and saliva.

**Fig. 1:**
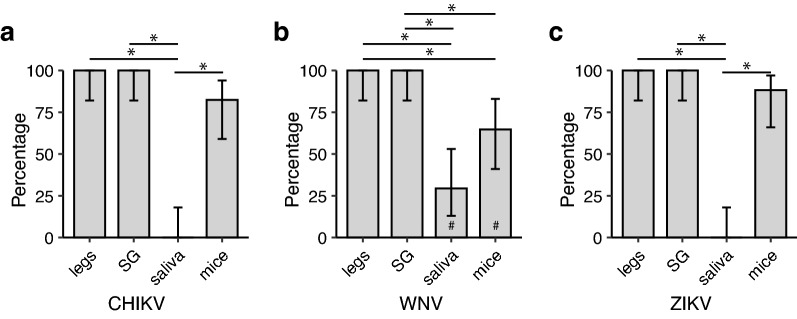
Transmission rate estimated for *Aedes aegypti* mosquitoes injected with three different arboviruses, based on sample type. **a** CHIKV (*N* = 17), **b** WNV (*N* = 18/^#^17), **c** ZIKV (*N* = 17). Error bars represent the 95% binomial confidence intervals. Asterisks (*) indicate significant comparisons based on a Fisher exact [23] test after Holm correction for multiple testing [24].* Abbreviations*: CHIKV, Chikungunya virus;* SG*, salivary glands; WNV, West Nile Virus; ZIKV, Zika virus

We first estimated transmission of three arboviruses (CHIKV, ZIKV, and WNV) by *Ae. aegypti.* All mosquito legs tested were positive for the corresponding injected virus, confirming dissemination (Fig. 1). The brain and/or hind limb from 82.4% (14/17) of suckling mice tested positive for CHIKV after being fed upon by infected *Ae. aegypti* (Fig. 1a). Examination of the proxies revealed a positivity rate of 100% (17/17) and 0% (0/17) for the salivary glands and saliva, respectively (Fig. 1a). Statistical analysis revealed no significant difference in the infection rates of SG and mice (*P* = 0.68) whereas the positivity of saliva collected by forced salivation significantly differed from the infection rates in mice (*P* < 0.001).

While *Ae. aegypti* is not considered a vector for WNV, studies have demonstrated its competency in the laboratory and, thus, it was utilized to further evaluate transmission proxy accuracy. Infected *Ae. aegypti* transmitted WNV to 61.1% of exposed mice (11/17; Fig. 1b). As with CHIKV, all SG were positive (100%, 18/18), yet the proportion of mosquitoes with WNV in saliva secretions was 27.8% (5/17) (Fig. 1b). Based on these findings, the results for the SG significantly overestimated transmission events when compared to the infection rates in mice (*P* = 0.03). Conversely, the percentage of positive saliva samples was lower than that of WNV-positive mice, but this difference was not statistically different (*P* = 0.17) (Fig. 1b).

*Aedes aegypti* transmitted ZIKV to 88.3% of exposed mice (15/17) (Fig. 1c), with all of the SG and none of the saliva secretions testing positive (Fig. 1c). Statistically, ZIKV transmission was better estimated from the positivity of SG (*P* = 1.00) than from positive saliva (*P* < 0.001).

**Fig. 2:**
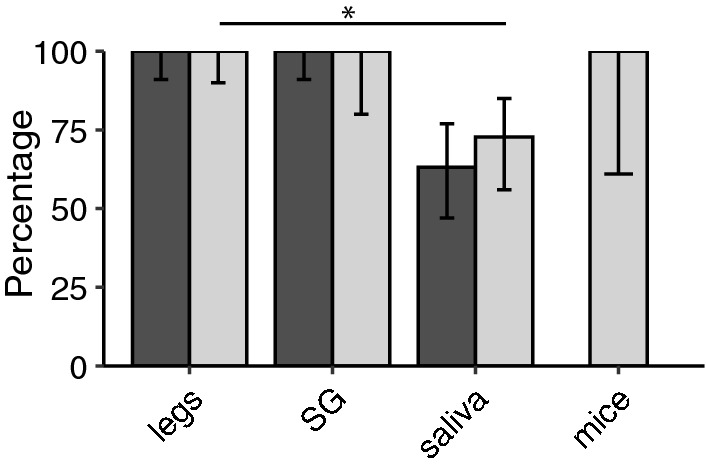
Transmission rate estimated for *Culex quinquefasciatus* mosquitoes injected with WNV, based on sample type at 7 dpi (dark-gray bars; *N* = 38) and 10 dpi (light-gray bars; *N* = 33^†^). ^†^SG were only dissected from 15 out of the 33 mosquitoes at 10 dpi and that only 6 mice were successfully exposed to WNV. Error bars represent the 95% binomial confidence intervals. Asterisks (*) indicate significant comparisons at 10 dpi based on a Fisher exact [23] test after Holm correction for multiple testing [24].* Abbreviations*: dpi, Days post-infection

Since *Ae. aegypti* is not the natural vector of WNV, we performed transmission assays on *Cx. quinquefasciatus* mosquitoes, a major vector of WNV in North America, inoculated intrathoracically. All *Cx. quinquefasciatus* with disseminated infection also had positive SG (100%; Fig. 2) at 7- and 10-days post-infection (dpi). Despite our efforts, we only recorded six out of 16 mosquitoes either probing or feeding on the mice. All mice exposed to WNV became infected (100%; Fig. 2). WNV was detected in 63.2% (24/38) and 72.7% (24/33) of the saliva samples 7 and 10 dpi, respectively (Fig. 2). Estimates of transmission rate using saliva were not significantly different from the transmission rate observed in suckling mice at dpi 10 (*P* = 1.00), nor were the estimates based on SG (*P* = 1.00). However, due to the low numbers of exposed mice, these results should be interpreted with caution.

**Fig. 3:**
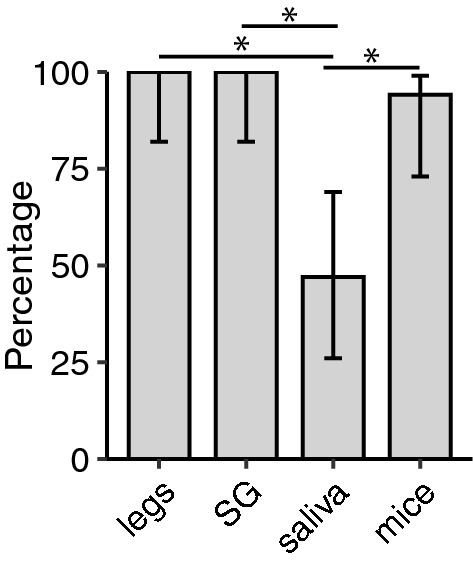
Transmission rate estimated for *Aedes triseriatus* mosquitoes injected with LACV based on sample type (*N* = 17). Error bars represent the 95% binomial confidence intervals. Asterisks (*) indicate significant comparisons based on a Fisher exact [23] test after Holm correction for multiple testing [24].* Abbreviations*: LACV, La Crosse virus

We further evaluated transmission proxy accuracy utilizing a member of the family Peribunyaviridae (LACV) and *Ae. triseriatus* mosquitoes. High transmission rates were observed in suckling mice fed upon by LACV-infected *Ae. triseriatus* (16/17, 94.1%). All SG of disseminated mosquitoes were positive (100%), while 47.1% (8/17) of the saliva samples were positive for LACV (Fig. 3). Positivity of SG better reflected LACV transmission to suckling mice than saliva (*P* = 1.00 and *P* = 0.0264, respectively) in this system.

### Influence of infection route on transmission proxies

**Fig. 4:**
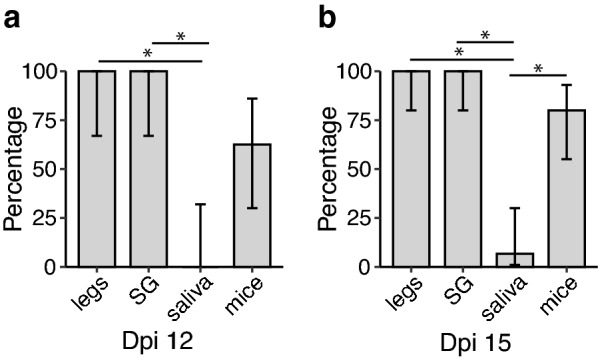
Transmission rate estimated for orally infected *Ae. aegypti* mosquitoes with CHIKV based on sample type after 12 dpi (*N* = 8) (**a**) and 15 dpi (*N* = 15) (**b**). Error bars represent the 95% binomial confidence intervals. Asterisks (*) indicate significant comparisons based on a Fisher exact [23] test after Holm correction for multiple testing [24]

Since IT inoculation of arboviruses bypasses the midgut barriers to infection and thus does not follow the natural infection route, we tested whether infection route influenced the accuracy of transmission proxies by orally infecting *Ae. aegypti* with CHIKV. The infection status and transmission capacity of mosquitoes fed with a CHIKV-infected blood meal were determined at 12 and 15 dpi. Only fully fed mosquitoes were used for the analysis. Infection rate, estimated from the presence of virus in bodies, was 52.9% (9/17) at 12 dpi and reached 100% by 15 dpi (18/18). All mosquitoes with CHIKV-positive legs had CHIKV-positive SG. Among the infected mosquitoes (positive bodies), 88.9% (8/9) developed disseminated infections at 12 dpi and 83.3% (15/18) at 15 dpi. From the suckling mice that were fed upon by a mosquito with a disseminated infection, 62.5% (5/8) became infected from mosquitoes with a 12-day EIP and 80% (12/15) from mosquitoes with a 15-day EIP (Fig. 4). The percentage of suckling mice that became infected by a mosquito was not statistically different from positivity based on legs or SG at 12 dpi (*P* = 0.6) or 15 dpi (*P* = 0.6724). No positive saliva was detected at 12 dpi, and only one sample was positive for CHIKV at 15 dpi. The positivity of saliva was not statistically different to that of suckling mice at day 12 EIP (*P* = 0.1025) but it was significantly different at day 15 EIP (*P* < 0.001).

### Accuracy of transmission proxies

**Fig. 5:**
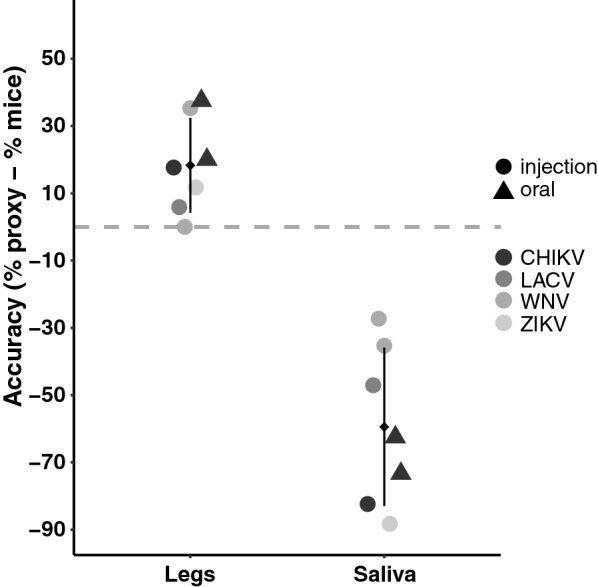
Accuracy of proxies relative to direct virus transmission estimates to suckling mice. Vertical axis shows the difference in the percentage of transmission estimated between the proxy and the mice infections. Different shades of gray represent the different viruses. Experiments from intrathoracic injection are indicated by the larger circles and oral infections by triangles. Mean and standard deviation of the proxy are indicated by the small diamond and attached vertical line, respectively

Across all vector-virus pairs assayed, SG infection status was identical to that of legs, independently of infection route (all pairwise comparisons *P* = 1.00). The presence of virus in legs (and thus of SG) more closely reflected direct transmission values to suckling mice, overestimating transmission by an average of 18.29 ± 14.09% (Fig. 5). In comparison, transmission estimated from virus positivity in saliva underestimated transmission by 59.43 ± 23.55% (Fig. 5). The difference in accuracy was significant (Welch t-test, *P* < 0.001) and the pattern was observed regardless of the mode of infection (IT inoculation vs oral feed).

## Discussion

Accurately assessing the ability of a mosquito population to transmit a pathogen is critical to determining its potential to serve as a disease vector. In the laboratory, this is achieved by performing forced salivation on individual mosquitoes, having mosquitoes feed on susceptible vertebrate hosts or assaying peripheral tissues, such as legs, heads and/or SG, for virus infection. In recent years, due to the cost and increasing restrictions on the use of vertebrates, forced salivation has become the accepted method for assaying transmission potential, despite a lack of empirical data demonstrating its accuracy at predicting transmission.

Forced salivation techniques lack standardization, and our data suggest that in our set-up, they misrepresent arboviral transmission to vertebrate hosts. In these techniques, mosquitoes may be anesthetized by cold, CO_2_ or triethylamine, following which their proboscises are immersed in one of many possible saliva collection diluents (sugar, virus media, FBS, or immersion oil). Smith et al. [27] reported no differences in Venezuelan equine encephalitis virus titers between saliva collected in mineral oil or fetal bovine serum (FBS) media. Miller et al. [20] consistently found the same was true for CHIKV and ZIKV but noted that saliva collected in mineral oil was more often positive for virus than that collected in FBS media [20]. Each of these variables can impact mosquito physiology and the salivation process in different ways and may account for observed inter-study variability. This was demonstrated in three studies examining *Ae. aegypti* transmission rates via forced salivation, in which 0% to approximately 45% of ZIKV-exposed mosquitoes were reported to have detectable virus in saliva following a 14-day EIP [28–30]. Such discrepancies may be attributed to differences between mosquito and virus strains, or suggest variation in forced salivation techniques. While the goal of this study was not to discriminate different forced salivation techniques, we did find that forced salivation in general underrepresents the transmission potential of an arbovirus when compared to feeding on live animals.

Direct feeding on laboratory animals is the gold standard for assessing arbovirus transmission because it most accurately recapitulates the biological and physiological conditions associated with natural transmission events. Previous studies have shown that suckling outbred mice are a reliable animal model for CHIKV, WNV, ZIKV and LACV [15–19], as they develop viremia and severe disease and mortality often accompany infection [31]. We are thus confident that the direct transmission rates we observed for these pathogens accurately reflect their transmission potential. Nevertheless, although direct mosquito feeding can be highly sensitive for assessing arbovirus transmission, it is dependent on the availability of a suitable animal model, limiting the utility of this approach for many arboviruses.

Alternative approaches to estimate virus transmission by mosquitoes involve testing head/SG tissue via PCR assay, immunofluorescence assay (IFA) or virus titration. In this study we examined SG tissue by PCR assay as another proxy for transmission and found that it overestimated direct transmission rates to mice, but that was closer to direct transmission rates than were those estimated from forced salivation. The positivity of SG was identical to that in legs in this system, despite our efforts to rinse the SG after dissection. A possible explanation is that contamination of the SG by the surrounding hemolymph may have inflated the number of positive SG samples recorded and that this parameter may not reflect an actual SG infection, but rather the attachment of virus particles to the exterior of this tissue. Had we performed IFA, infection rates from SG may have been in closer agreement with the direct feeding results. Nevertheless, these results suggest that dissemination rate (as measured from the legs) may be a good proxy for transmission, without the need of time-consuming and technically challenging dissection of SG.

The poor performance of saliva collected via capillary tubes as proxy for transmission was observed regardless of the mode of infection. While we are aware that infection via IT injection does not reflect a natural route of infection, the focus of this work was to measure the effectiveness of different methods to estimate transmission rate once infection becomes established. We relied on IT inoculations for the bulk of the studies to increase the sample size of our treatments and minimize the use of vertebrates. By using IT injections, we were able to bypass any potential midgut infection and/or escape barriers, thus ensuring that all the mosquitoes tested had a disseminated infection. We further investigated whether our results were dependent on the mode of infection by performing both IT and oral infections with *Ae. aegypti* and CHIKV. A longer EIP was considered for the oral infection, since IT inoculation is known to reduce EIP [15, 27, 29, 30]. The data from the oral infections supports our findings that forced salivation methods significantly underestimate transmission in this system. In these experiments, detection of virus in saliva proved more difficult in *Ae. aegypti* than for the other two species tested, regardless of the virus. Studies have reported low transmission for CHIKV and ZIKV in the *Ae. aegypti* ORL strain, relative to other strains [32, 33], and we cannot discard the possibility that our results may reflect, in part, an overall lower competence level of this particular colony.

The lack of accuracy of forced salivation to reflect arboviral transmission likely results from low viral saliva titers during the early stages of transmission that fall below assay detection thresholds [20, 22], within-population variability in viral expectoration [22] further influenced by differences in vector-virus pairings [27] and/or the possibility that mosquitoes may re-ingest their saliva during forced salivation, as they do during an artificial blood feed [20]. Moreover, there is evidence that SG respond differently following acquisition of a sugar meal and a blood meal [39]. Consequently, saliva collected during forced salivation may not accurately mimic the saliva expectorated during blood-feeding. The interplay of all these variables, further amplified by the lack of standardization of forced salivation techniques across laboratories caution against the use of saliva as a proxy for transmission without prior knowledge of the system investigated.

## Conclusions

Our results suggest that virus positivity of legs or SG (dissemination rate) are overall more accurate predictors of arboviral transmission than detection of virus from saliva collected using the commonly implemented forced salivation technique and are subject to less technical variation. However, a leg-only approach for estimating transmission rates may not be appropriate for all studies. When examining the competency of a new virus–vector pairing, multiple approaches for estimating transmission rate should be implemented to explore the possibility that a SG infection barrier or escape barrier exists. Moreover, the use of legs as transmission proxy should be utilized judicially when examining EIP. Based on the kinetics of infection, the hemolymph/legs will become infected prior to transmission; therefore, studies assessing transmission through the use of legs alone may report shortened EIPs.

## Supplementary Information

**Additional file 1: Text S1.** Titration of viral stock solutions. **Table S1.** Sequence of primers and probes (5’ to 3’) used for viral detection in the RT-PCR assay. **Table S2.** Limit of detection of RT-PCR assay. **Figure S1**. RT-PCR standard curves for chikungunya (a), Zika (b), La Crosse (c) and West Nile (d) viruses.

## Data Availability

All data generated or analyzed during this study are included in this published article and its supplementary information files.

## Abbreviations

CHIKV: Chikungunya virus
EIP: Extrinsic incubation period
IIP: Intrinsic incubation period
IT: Intrathoracic inoculation
LACV: La Crosse virus
ORL: *Aedes aegypti* Orlando strain
PFU: Plaque-forming units
SG: Salivary glands
WNV: West Nile virus
ZIKV: Zika virus
