# Supplementary material for: Saliva collection via capillary method may underestimate arboviral transmission by mosquitoes
Source: Parasit Vectors. 2022 Mar 24;15:103. doi: 10.1186/s13071-022-05198-7 (PMC8944160; doi:10.1186/s13071-022-05198-7)
Supplement: Supplementary file 1 — Additional file 1: Text S1. Titration of viral stock solutions. Table S1. Sequence of primers and probes (5’ to 3’) used for viral detection in the RT-PCR assay. Table S2. Limit of detection of RT-PCR assay. Figure S1. RT-PCR standard curves for chikungunya (a), Zika (b), La Crosse (c) and West Nile (d) viruses. [file 13071_2022_5198_MOESM1_ESM.docx]

**Supplementary methods**

*Titration of viral stock solutions*

The concentration of stock virus solutions (PFU: plaque forming units/mL) was determined via plaque assay. Briefly, plaque titrations were performed in six-well plates. Serial 10-fold dilutions of each virus isolate were absorbed onto confluent Vero cell monolayers for 45 min at 37°C, 5% CO2. Supernatant was then removed and cell monolayers were overlaid with 2 ml of 1% methylcellulose in nutrient medium (10% fetal bovine serum, 100 U ml^−1^ penicillin, 100 mg ml^−1^ streptomycin, L-glutamine, 25 mg/ml, sodium bicarbonate, and 5.2ml of non-essential amino acids). Monolayers were incubated at 37°C, 5% CO2 for 4 days (6 days for LCV). Plaques were scored after a 2 hrs fixation with a 7.4% formaldehyde solution. RT-PCR standard curves were constructed in triplicate by diluting stock virus solutions (of known concentration) sequentially 10-fold (Figure S1). Results were analyzed and plotted in the BioRad CFX Maestro^TM^ Software for Mac 1.1. v.4.1.2. using a baseline of 100.

**Table S1:** Sequence of primers and probes (5’ to 3’) used for viral detection in the RT-PCR assay.

| **Virus** | **Primer 1** | **Primer 2** | **Probe** | **Reference** |
| --- | --- | --- | --- | --- |
| CHIKV | TCACTCCCTGTTGGACTTGATAGA | TTGACGAACAGAGTTAGGAACATACC | AGGTACGCGCTTCAAGTTCGGCG | Lanciotti et al. (2007) |
| WNV | AAGTTGAGTAGACGGTGCTG | AGACGGTTCTGAGGGCTTAC | CTCAACCCCAGGAGGACTGG | Gill (2012) |
| ZIKV | CCGCTGCCCAACACAAG | CCACTAACGTTCTTTTGCAGACAT | AGCCTACCTTGACAAGCAGTCAGACACTCAA | Lanciotti et al. (2008) |
| LCV | TATAAAAGCCTAAGAGCTGCCAGAGT | GACCAGTACTGCAGTAATTATAGACAAT | TGTGCAAGTCGAAAGGGCCTGCA | Lambert et al. (2005) |

**Table S2:** Limit of detection of RT-PCR assay.

| **Virus** | **Lower Ct [A]** | **Higher Ct [B]** | **log10 PFU (A-B)** |
| --- | --- | --- | --- |
| CHIKV | 13.9 ± 0.333 | 34.7 ± 3.005 | 5.4 - 0.4 |
| WNV | 15.8 ± 0.329 | 36.7 ± 0.304 | 7.7 - 2.7 |
| LCV | 16.9 ± 0.195 | 36.3 ± 0.460 | 7.5 - 2.5 |
| ZIKV | 22.3 ± 0.747 | 37.7 ± 2.550 | 6.2 - 2.2 |

PFU: plaque forming units per mL

**Figure S1**: RT-PCR standard curves for A) Chikungunya, B) Zika, C) La Crosse, and West Nile virus.

**Supplementary references:**

Lanciotti, R.S., Kosoy, O.L., Laven, J.J., Panella, A.J., Velez, J.O., Lambert, A.J. and Campbell, G.L., 2007. Chikungunya virus in US travelers returning from India, 2006. *Emerging infectious diseases*, *13*(5), p.764.

Lanciotti, R.S., Kosoy, O.L., Laven, J.J., Velez, J.O., Lambert, A.J., Johnson, A.J., Stanfield, S.M. and Duffy, M.R., 2008. Genetic and serologic properties of Zika virus associated with an epidemic, Yap State, Micronesia, 2007. *Emerging infectious diseases*, *14*(8), p.1232.

Gill, B. S. (2012). Epidemiology of dengue in Malaysia from 2005 - 2010 and factors contributing to its emergence*.* (Doctoral thesis, The University of Western Australia).

Lambert, A.J., Nasci, R.S., Cropp, B.C., Martin, D.A., Rose, B.C., Russell, B.J. and Lanciotti, R.S., 2005. Nucleic acid amplification assays for detection of La Crosse virus RNA. *Journal of clinical microbiology*, *43*(4), pp.1885-1889.
